# Supplementary material for: Effect of GR24 on the growth and development of licorice under low phosphorus stress
Source: PeerJ. 2024 Nov 26;12:e18546. doi: 10.7717/peerj.18546 (PMC11606326; doi:10.7717/peerj.18546)
Supplement: Supplemental Information 1 — Specific chlorophyll calculation formulas and detection methods for medicinal ingredients. [file peerj-12-18546-s001.docx]

1. Calculation formula for chlorophyll a and b

Chlorophyll a content (mg/g)=(12.27 * A663-2.59 * A645) * Vs/1000w

Chlorophyll b content (mg/g)=(22.88 * A645-4.67 * A663) * Vs/1000w

Vs - total volume of extraction solution, ml;

W - Quality of fresh licorice leaves, g.

1. Specific methods and parameters for HPLC determination of medicinal ingredients

The dried roots of *Glycyrrhiza uralensis* were crushed, passed through a 40 mesh, sieve， and weighed and set aside. The contents of glycyrrhizic acid, glycyrrhetic acid, Lsoliquirtigenin, Lsoliquirtin, Glabridin, Liquirtigenin, and Liquirtin were determined using an ACQUITY UPLC ultra-high-performance liquid chromatography (UHPLC), a triple quadruple quadrupole tandem mass spectrometer (XEVO TQ-S), and a MassLynx workstation (Waters Corporation, USA). The chromatographic column was a Waters ACQUITY UPLC BEH C_18_ column (50 mm × 2.1 mm, 1.7 μm). The optimized mobile phases were A (0.1% formic acid-water), B (acetonitrile) at a flow rate of 0.3 mL/min, the column temperature: 40 ℃, the injection volume of 1 μL, and the gradient elution in the order of:

Table1 Gradient elution

| Time (min) | Flow rate (mL/min) | 0.1% formic acid-water (%) | Acetonitrile (%) |
| --- | --- | --- | --- |
| 0 | 0.3 | 80 | 20 |
| 1.0 | 0.3 | 80 | 20 |
| 1.5 | 0.3 | 50 | 50 |
| 2.0 | 0.3 | 0 | 100 |
| 3.5  3.8 | 0.3  0.3 | 0  80 | 100  20 |
| 5.0 | 0.3 | 80 | 20 |

Mass spectrometry conditions the Ion source was an electrospray ionization (ESI) source, and the content determination was carried out in multiple reaction detection mode (MRM), desolvation gas temperature: 450 ℃; source temperature: 150 ℃; desolvation gas flow rate: 800 L/h; conical pore gas: 150 L/h; capillary voltage: 3000 V. The daughter ions of the BZA were obtained based on the optimization of the collision-induced dissociation of their parent ions, which were used for the quantitative analysis. The ion pairs, cleavage voltages, and collision energies for quantitative analysis were as follows:

Table2 Mass spectrometry parameters for quantitative components

| ingredient | t(min) | ionization mode | Parent ion MS (m/z) | Sub-ion MS^2^ (m/z) | Cone hole voltage/V | Crash energy/eV |
| --- | --- | --- | --- | --- | --- | --- |
| Glycyrrhizic acid | 4.84 | ESI^+^ | 823.13 | 453.1* | 12 | 22 |
|  |  |  |  | 647.1 | 12 | 12 |
| Liquirtigenin | 3.32 | ESI^+^ | 257.1 | 136.9* | 92 | 24 |
|  |  |  |  | 146.9 | 92 | 18 |
| Liquirtin | 4.59 | ESI^+^ | 256.9 | 136.8* | 2 | 24 |
|  |  |  |  | 146.9 | 2 | 18 |
| Lsoliquirtigenin | 1.89 | ESI^-^ | 416.9 | 254.9* | 52 | 20 |
|  |  |  |  | 134.9 | 52 | 30 |
| Lsoliquirtin | 3.06 | ESI^-^ | 416.9 | 254.9* | 52 | 20 |
|  |  |  |  | 134.9 | 52 | 30 |
| Glycyrrhetic acid | 6.43 | ESI^-^ | 469.1 | 355.0* | 100 | 44 |
|  |  |  |  | 409.1 | 100 | 46 |
| Glabridin | 6.06 | ESI^-^ | 322.9 | 134.9* | 2 | 18 |
|  |  |  |  | 200.9 | 2 | 24 |

Note: * represents quantitative ion.

Accurately weigh 2mg of each standard, respectively, in a 10mL volumetric flask, and the methanol was fixed to obtain a standard stock solution with a mass concentration of 200 μg/mL. Precisely aspirate the reserve solution of 7 controls respectively, and prepare the methanol solution into the control solution with mass concentrations of 10, 100, 250, 500, and 1000 ng/mL. 1.5 mL was pipetted into an Agilent injection vial, and 1 μL was injected into the vial respectively. The working curve was plotted with the peak area Y as the vertical coordinate, the mass concentration (x, ng/mL) as the horizontal coordinate, and the correlation coefficient*, R*^2^ above 0.9960. The results showed that there was a good linear relationship between the concentration of the control and the peak area in the range of 9.3-1000.4 ng/mL.

Table3 Regression equation and correlation coefficient of chemical components of *Glycyrrhiza uralensis* Fisch

| chemical compound | regression equation | Correlation coefficient R^2^ | Linear range ng/ml |
| --- | --- | --- | --- |
| Glycyrrhizic acid | Y=3150.8*X+14239.2 | 0.9992 | 9.3 - 1000.4 |
| Liquirtigenin | Y=720.3*X+1644.0 | 0.9985 | 9.0 - 969.1 |
| Liquirtin | Y=25676.3*X+26145.2 | 0.9999 | 1.0 - 498.8 |
| Lsoliquirtigenin | Y=516.9*X+3821.2 | 0.9991 | 1.3 - 1001.2 |
| Lsoliquirtin | Y=626.8*X+859.7 | 0.9981 | 0.8 - 961.8 |
| Glycyrrhetic acid | Y=425.6*X+109.5 | 0.9998 | 0.9 - 992.4 |
| Glabridin | Y=156.0*X+78.3 | 0.9960 | 0.6 - 954.0 |

The powder of each treatment group was accurately weighed into three portions of 0.200 g each, which were placed in 10 mL centrifuge tubes, and each of them was added with 5 mL of methanol (chromatographic purity) as the extraction solution, and extracted superficially for 2 h with an ultrasonic extractor with a power of 300 W (KQ-300E, Kunshan Ultrasonic Instrument Co., Ltd., China), and then centrifuged for 15 min with a high-speed centrifuge (12,000 r/min), and then the upper supernatant was taken. After centrifugation for 15 min with a high-speed centrifuge (12000 r/min), the supernatant was filtered through a microporous membrane with a pore size of 0.25 μm and poured into an Agilent injection vial with an injection volume of 1 μL, and the determination was repeated three times for each sample.

1. Sequencing quality assessment form

Table3 Sequencing data results

| Samples Name | Clean reads | Clean bases | GC Content |
| --- | --- | --- | --- |
| A1 | 22,952,611 | 6,872,115,873 | 44.70% |
| A2 | 19,426,608 | 5,820,601,375 | 44.74% |
| A3 | 21,880,694 | 6,553,263,471 | 44.83% |
| B1 | 21,245,886 | 6,363,773,413 | 44.21% |
| B2 | 23,206,194 | 6,950,027,201 | 45.01% |
| B3 | 21,746,087 | 6,511,936,937 | 43.91% |
| D1 | 21,223,568 | 6,353,161,680 | 44.37% |
| D2 | 20,982,722 | 6,265,701,629 | 43.51% |
| D3 | 22,801,827 | 6,814,059,838 | 44.37% |
| E1 | 20,581,537 | 6,161,220,909 | 44.52% |
| E2 | 20,322,757 | 6,084,402,825 | 43.95% |
| E3 | 21,462,333 | 6,416,074,397 | 43.51% |
| G1 | 24,457,003 | 7,298,495,974 | 43.99% |
| G2 | 29,416,246 | 8,729,915,104 | 43.87% |
| G3 | 27,500,000 | 8,200,121,174 | 43.86% |
| H1 | 25,544,621 | 7,646,695,951 | 44.99% |
| H2 | 21,992,193 | 6,582,249,795 | 43.76% |
| H3 | 23,310,473 | 6,975,484,972 | 43.26% |

Table 4 Sequencing quality assessment

| Sample Name | Total Reads | Mapped Reads | % ≥ Q30 |
| --- | --- | --- | --- |
| A1 | 45,905,222 | 39,000,533 (84.96%) | 96.64% |
| A2 | 38,853,216 | 33,218,927 (85.50%) | 98.02% |
| A3 | 43,761,388 | 38,489,298 (87.95%) | 97.13% |
| B1 | 42,491,772 | 37,980,622 (89.38%) | 97.49% |
| B2 | 46,412,388 | 42,142,991 (90.80%) | 96.61% |
| B3 | 43,492,174 | 36,359,845 (83.60%) | 96.65% |
| D1 | 42,447,136 | 35,925,303 (84.64%) | 96.67% |
| D2 | 41,965,444 | 35,450,665 (84.48%) | 96.83% |
| D3 | 45,603,654 | 35,998,406 (78.94%) | 96.90% |
| E1 | 41,163,074 | 34,735,163 (84.38%) | 96.62% |
| E2 | 40,645,514 | 32,985,637 (81.15%) | 98.09% |
| E3 | 42,924,666 | 36,394,007 (84.79%) | 97.93% |
| G1 | 48,914,006 | 42,162,916 (86.20%) | 96.70% |
| G2 | 58,832,492 | 51,712,421 (87.90%) | 96.80% |
| G3 | 55,000,000 | 48,230,974 (87.69%) | 97.97% |
| H1 | 51,089,242 | 36,687,309 (71.81%) | 95.25% |
| H2 | 43,984,386 | 38,126,116 (86.68%) | 96.97% |
| H3 | 46,620,946 | 38,533,581 (82.65%) | 95.95% |
